# The Association Between Bedtime Procrastination, Sleep Quality, and Problematic Smartphone Use in Adolescents: A Mediation Analysis

**DOI:** 10.5152/eurasianjmed.2024.23379

**Published:** 2024-02-01

**Authors:** Abdullah Bozkurt, Esen Yıldırım Demirdöğen, Mehmet Akif Akıncı

**Affiliations:** Department of Child and Adolescent Psychiatry, Atatürk University Faculty of Medicine, Erzurum, Turkey

**Keywords:** Adolescents, sleep quality, bedtime procrastination, problematic smartphone use

## Abstract

**Background:**

This study investigated the relationships between smartphone use, bedtime procrastination, and adolescent sleep quality. Specifically, the study investigated whether bedtime procrastination mediates the relationship between problematic smartphone use (PSU) and sleep quality.

**Methods:**

A sample of 245 adolescents aged 13-18 participated in the study. The Smartphone Addiction Scale—Short Version, Bedtime Procrastination Scale, and Adolescent Sleep–Wake Scale were used to assess PSU signs, bedtime procrastination, and sleep quality, respectively. In addition, smartphone usage habits were assessed through a purpose-built questionnaire.

**Results:**

It was found that 46.9% of the adolescents had PSU signs, they used smartphones for an average of 2.8 hours per day, 90.6% used smartphones in bed, they used smartphones in bed for an average of 4.9 days per week, and the average duration of smartphone use in bed was 2.3 hours per day. Correlation analyses showed that poor sleep quality was positively associated with the duration of smartphone use in bed, number of smartphone activities, PSU signs, and bedtime procrastination. The relationship between PSU and sleep quality is mediated by bedtime procrastination, according to mediation analysis.

**Conclusion:**

This study demonstrated the negative effect of smartphone use on sleep quality in adolescents. The results also indicate that bedtime procrastination may play a mediating role between PSU and sleep quality. Thus, interventions such as sleep hygiene targeting bedtime procrastination may improve adolescents’ sleep quality with PSU signs.

**Main Points**

- This study demonstrated the negative effect of smartphone use on sleep quality in adolescents.
- The results indicate that bedtime procrastination may play a mediating role between problematic smartphone use (PSU) and sleep quality.
- Interventions such as sleep hygiene targeting bedtime procrastination may improve adolescents’ sleep quality with PSU signs.

## Introduction

In recent years, the increase in sleep problems and the decline in sleep quality have emerged as significant issues impacting public health and the development of adolescents.^1^ Sleep plays a critical role in young people’s physical, mental, and emotional development. Sleep disorders lead to severe challenges in fundamental areas such as learning, memory, emotional regulation, and social functioning in adolescents.^2^ Various factors can disrupt sleep patterns and negatively affect sleep quality in this age group.

The rise in poor-quality sleep among adolescents is attributed to a wide range of factors. Technological advancements and the proliferation of digital media use are significant factors that considerably impact adolescents’ sleep patterns and quality. Smartphones, tablets, and other screen-based devices, mainly used before bedtime, can adversely affect sleep.^3^ Additionally, increased academic pressures, social stresses, and biological changes during puberty are other significant factors affecting sleep quality.^4^ Particularly, the social isolation, education disruptions, and general lifestyle changes experienced during the coronavirus disease 2019 pandemic have profoundly and extensively impacted adolescents’ sleep patterns. The closure of schools during the pandemic and the shift to remote learning models significantly altered their daily routines and sleep patterns.^5^ The reduction in social interaction and limited opportunities for physical activity led to increased levels of anxiety and stress among adolescents, indirectly contributing to the deterioration of sleep quality.^6^ Moreover, increased screen time and late-night digital device use during the pandemic have further disrupted young people’s sleep patterns.^7^

The role of mobile phone screen time in adolescents is significant, and there are concerns about its impact on cognitive health, mental well-being, and physical health.^8,9^ Problematic smartphone use (PSU) among young people has become an increasingly worrying issue in recent years. Problematic smartphone use is a pattern of smartphone usage that includes dysfunctional elements such as anxiety when not using the smartphone or neglecting other activities due to excessive usage.^10^ Problematic smartphone use leads to adverse outcomes among children and adolescents, including reduced academic achievement, decreased learning efficiency, physical symptoms, sleep disturbances, and psychopathological issues.^11^ The use of electronic media (e.g., smartphones) in the evenings, particularly among adolescents and young adults, is continuously increasing, significantly impacting their sleep quality and patterns.^12^ These impacts are primarily mediated through various biological and behavioral mechanisms, such as disruption of circadian rhythms, increased arousal, and bedtime procrastination.

The sleep/wake rhythm is known as the circadian rhythm, regulated by the hormones melatonin and cortisol. Findings indicate that light-emitting diode (LED) backlit screens, like smartphones, emit more intense light than other types of screens, which poses a significant concern for human circadian physiology.^13,14^ Excessive use of smartphones before bedtime is one of the factors contributing to sleep disturbances through increased arousal levels. The interactive nature of smartphones, offering numerous stimuli from social networks, games, and news, can lead to a state of high mental alertness, contrary to the calm required for sleep, by triggering cognitive arousal.^15,16^ Research has demonstrated a relationship between increased cognitive arousal and reduced sleep quality.^17^

Bedtime procrastination is not going to bed at the intended time despite no external circumstances preventing it.^18^ There are several reasons for people’s behavior to delay bedtime. Strategic procrastination is the act of postponing sleep to avoid damaging emotions associated with sleep or to accumulate sleep pressure in cases of difficulty falling asleep. Conscious bedtime delay involves deliberately postponing sleep time to complete additional tasks or to allocate time for oneself after a busy day. In unconscious bedtime procrastination, the individual becomes so engrossed in a task that they lose track of time.^19^ A study on university students has shown that bedtime procrastination can strongly determine the severity and prevalence of poor sleep quality.^17^ It has been found that individuals with lower self-regulation skills, which refer to the ability to manage their behaviors, emotions, and thoughts in order to achieve goals, are more prone to postpone bedtime. The relationship between low self-regulation in individuals and behavioral addictions, such as excessive phone use, has also been established.^20,21^ Furthermore, neurocognitive research suggests that addictions may harm brain regions related to an individual’s self-regulation abilities.^22^ Therefore, examining the relationships between PSU and bedtime procrastination in adolescents is necessary due to shared cognitive interactions like self-regulation and the potential risks of smartphone use as a behavior for sleep delay.

Despite studies examining the mediating effect of variables such as self-regulation, bedtime procrastination, and fear of missing out on sleep quality on the effect of smartphone use in the adult age group, there are not enough studies in adolescence, which is a critical period for mental and physical development.^23-25^ This study aimed to learn the frequency of smartphone use and types of activities in bed before sleep, the relationships, and sex differences between PSU, bedtime procrastination, and sleep quality in Turkish adolescents. In addition, the mediating effect of bedtime procrastination between PSU and sleep quality was also examined. We hypothesized that PSU signs would have a positive correlation with poor sleep quality and that bedtime procrastination would mediate the link between PSU and poor sleep quality.

## Material and Methods

### Participants

This study consisted of randomly selected adolescents aged 13-18 in Erzurum, Turkey. The sample consisted of 245 adolescents who participated in the study and completed the questionnaire. This study was approved by Ethics committee of Atatürk University (Approval No: B.30.2ATA.0.01.00/748, Date: 26.10.23). The study complied with the principles of the Declaration of Helsinki. Written informed consent was received from all participants and their parents. The research was conducted between October 30, 2023, and December 10, 2023.

### Measures

#### Problematic Smartphone Use

Smartphone Addiction Scale-Short Version (SAS-SV) was used to evaluate PSU.^10^ Smartphone Addiction Scale-Short Version is a 6-point Likert-type scale consisting of 10 items. The total score obtained from the scale varies between 10-60 points. As the total score of SAS-SV increases, the risk of PSU increases. The scale provided valid and reliable measurements in a study conducted on adolescents in Turkish culture, and the cut-off score was 29.5 for both boys and girls.^26^

#### Bedtime Procrastination

The bedtime procrastination (BP) is a 9-item self-report scale assessing bedtime procrastination behavior.^18^ The participants rate these questions on a 5-point Likert scale ranging from 1 (never) to 5 (always). A higher score on the BP scale indicates more bedtime procrastination. Dinç et al^27^ conducted the Turkish adaptation of the scale.

#### Sleep Quality

The Adolescent Sleep–Wake Scale (ASWS) was used to evaluate sleep quality.^28^ The 10-item short form of ASWS used in this study was developed by Essner et al.^29^ A higher score indicates better sleep quality. The Turkish ASWS was found to be a valid and reliable instrument by the authors of this study.^30^

#### Smartphone Usage Behavior

In addition to standard tests, a purpose-built questionnaire was utilized to estimate daily smartphone usage and the duration of using other technological devices (such as tablets, computers, televisions, and gaming consoles), categorized into time intervals (0-1 hour, 1-2 hours, 2-3 hours, 3-4 hours, 4-5 hours, more than 5 hours). The questionnaire also inquired about smartphone usage in bed (yes/no), the frequency of pre-sleep smartphone use per week while in bed, the average duration of this usage (0-15 minutes, 16-30 minutes, 31-60 minutes, or more than 60 minutes), and finally, the types of activities performed on smartphones in bed (calling, messaging, browsing the internet, using social networks (Facebook, Instagram, TikTok, etc.), watching videos, playing games, or others).

### Statistical Analysis

Statistical analysis was performed using SPSS 26, (IBM SPSS Corp.; Armonk, NY, USA). Descriptive statistics were calculated for sociodemographic variables and study variables. Possible differences between girl and boy adolescents regarding study results were evaluated using an independent sample *t*-test. Pearson correlations were used to analyze the relationships between the main variables. The effect size was calculated according to Cohen’s *d* statistic. The PROCESS macro (version 4.2 for SPSS IBM SPSS Corp.; Armonk, NY, USA.) was employed in this study to assess mediation and investigate the mediating role of bedtime procrastination in the PSU-sleep quality relationship. We analyzed the relationship between PSU and bedtime procrastination, bedtime procrastination and sleep quality, and the direct impact of PSU on sleep quality. Furthermore, an analysis was conducted on the indirect impact of PSU on sleep quality via bedtime procrastination. In calculating the coefficients of direct and indirect effects, confidence intervals of 95% were utilized.

## Results

Our study included 245 adolescents between the ages of 13-18, 100 girls and 145 boys. Adolescents used smartphones for an average of 2.8 hours per day. Smartphone use in bed was present in 93.5% of adolescents. When it was analyzed how many days of the week they used smartphones in bed, it was determined that 6.5% of the adolescents did not use smartphones in bed at all, 13.1% used smartphones in bed for 1 day, 7.3% for 2 days, 6.9% for 3 days, 5.3% for 4 days, 1.2% for 5 days, 3.7% for 6 days, and 55.9% for 7 days. When analyzing the duration of smartphone use in bed, it was found that 6.5% of teens never used smartphones in bed, 22.4% used smartphones for less than 15 minutes, 29% used smartphones for 16-30 minutes, 18% used smartphones for 31-60 minutes, and 24.1% used smartphones for more than 60 minutes. When analyzing the activities performed with the smartphone in bed, it was found that 35.9% of the adolescents made phone calls, 53.5% texted, 76.7% accessed social media sites, 51.4% browsed the web, 68.2% watched videos, 38% played games, and 25.7% performed other activities. Adolescents were divided into 2 groups, those with and without PSU, according to the cutoff point of the SAS-SV. Of the adolescents, 46.9% had pathological smartphone use. Descriptive information about adolescents is given in Table 1.

Adolescents used smartphones in bed 4.89 ± 2.63 days a week. They used an average of 3.49 ± 2.11 activities while using a smartphone in bed. The sleep time delay scale score was 26.51 ± 7.34. The PSU scale score was 29.61 ± 11.92. Sleep quality scale scores were 3.15 ± 0.74. These descriptive data of adolescents are given in Table 2.

In the analyses conducted to determine whether the descriptive data differed between sexes, it was found that technological devices other than smartphones were used more by boys. There was no difference between sexes in terms of other data. The data of the comparisons between the sexes are presented in Table 3.

Correlation analyses showed that sleep quality was negatively associated with the duration of smartphone use in bed, the number of activities conducted on the phone, PSU symptoms, and bedtime procrastination. The results of other correlation analyses are presented in Table 4.

**Figure 1.**
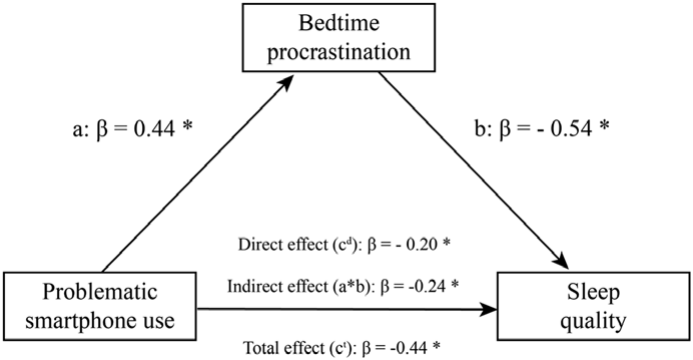
The mediating effect of bedtime procrastination on the relationship between problematic smartphone use and sleep quality in adolescents.

There is a statistically significant effect between PSU and bedtime procrastination (a: *β* = 0.44, *P* < .001) and between bedtime procrastination and sleep quality (b: *β* = −0.54, *P* < .001). The direct effect of PSU on sleep quality (*β* = −0.20, *P* < .001) and the indirect effect (the effect of PSU on sleep quality through bedtime procrastination) were found to be significant (*β* = −0.24, 95% CI (−0.02, −0.10)). Finally, a significant overall effect was found between PSU and sleep quality (*β* = −0.44, *P* < .001). These results indicate that bedtime procrastination has a partial mediating effect on the impact of PSU on sleep quality (the direct effect c^d^ is significant). Mediation analysis results are presented in Table 5 (Figure 1).

## Discussion

This study examined the daily smartphone usage duration, and frequency, duration, and type of activities of smartphone use at bedtime among adolescents, and sex differences in these factors and the relationships between PSU, bedtime procrastination, and sleep quality. In addition, the mediating effect of bedtime procrastination between PSU and sleep quality was evaluated.

This study found that 46.9% of adolescents exhibited signs of PSU. On average, they used smartphones for 2.8 hours per day and other technological devices for about 2 hours daily. Furthermore, 93.5% used a smartphone in bed. Smartphone use in bed averaged 2.3 hours per day and 4.9 days per week. The most frequent in-bed smartphone activities were using social network applications (76.7%), followed by watching videos (68.2%) and messaging (53.5%). Additionally, 64.9% of adolescents engaged in 3 or more activities on their smartphones while in bed. Correlation analyses showed that sleep quality was negatively associated with the duration of smartphone use in bed, the number of activities conducted on the phone, PSU symptoms, and bedtime procrastination. While boy adolescents used other technological devices more frequently, no significant sex differences were found in other variables. Finally, the mediation analysis revealed that bedtime procrastination had an indirect effect on the relationship between PSU and sleep quality.

The prevalence of PSU among adolescents has become an increasingly concerning issue in recent years. During the pandemic, the imposed social isolation provided children and adolescents with more opportunities and time to use digital devices for educational and entertainment purposes. Additionally, the pandemic period has led to widespread psychological problems, which in turn have increased the risks for digital addictions. Globally, the prevalence of PSU among young people was reported at 23.3% before the pandemic; however, this rate notably increased to 34.5% during the pandemic period.^31,32^ This study reveals that 46.9% of adolescents display symptoms of PSU, suggesting a continuation of the upward trend during the pandemic period. It is noteworthy in this research that while the average daily smartphone usage among adolescents is 2.8 hours, 2.3 hours of this usage occurs in bed. This study was conducted during the school term; hence, school occupies most of the day for adolescents. Restrictions on smartphone use in schools, after-school homework, additional tasks, and family supervision might limit adolescents’ mobile phone use during the day. The excessive time adolescents spend on smartphones in bed could be attributed to the lowest levels of family supervision and self-regulation due to fatigue occurring at bedtime.

Research on sex differences in technological addictions indicates a higher prevalence among boys, though there are also studies reporting no significant differences between sexes.^33,34^ A multi-center study found that boy students were more inclined towards internet and online gaming addiction, while girl students showed a higher tendency for addiction to online social networks.^35^ In this study, the increased usage duration of other technological devices among boy adolescents can be explained by activities such as gaming on consoles and computers and internet browsing. Although not statistically significant, the observation that girl adolescents exhibit more symptoms of PSU and spend more time using smartphones than their boy counterparts supports the literature, indicating a preference among girls for using technology primarily for social communication purposes.

Research has shown that adolescents with elevated levels of smartphone addiction are more likely to experience inadequate sleep.^36,37^ Excessive mobile phone use before bedtime not only delays sleep onset but may even replace sleep duration.^38^ Consistent with previous studies, this research has found a positive correlation between symptoms of PSU and poor sleep quality and bedtime procrastination. Furthermore, there is a positive correlation between total smartphone and technology usage times before bedtime and throughout the day and the practice of delaying bedtime. This finding is supported by a meta-analysis that suggests individuals who consume more electronic media are more prone to higher levels of bedtime procrastination behavior.^39^ As adolescence is a period sensitive to psychological risks, replacing sleep time with mobile phone use may exacerbate these risks.

Digital social networking services are among the most commonly used applications. Adolescents use social networks to fulfill social needs such as communication, entertainment, and peer interaction. Studies have reported a significant association between PSU and the use of social networks.^24,40^ Camerini et al. reported that adolescents engaged in online social entertainment activities tend to spend more time on these activities, allocate less time to sleep, and exhibit significantly higher levels of PSU over time.^41^ In our study, the predominance of social network use among adolescents using smartphones in bed can be attributed to the tendency to use social media through smartphones to link with others during the pandemic.^25^ The pleasure-oriented nature of social media applications and factors like adolescents’ low self-control pose risks for healthy sleep in this demographic. Furthermore, it has been found that smartphone usage varieties such as watching videos, playing games, and talking on the phone are also positively associated with PSU.^41-43^ Our research observed that as the number of smartphone activities in bed increases, there is greater bedtime procrastination and lower sleep quality. Restricting smartphone use activities before bedtime or preferring activities such as listening to relaxing music can reduce the risks of sleep problems.

This study demonstrates that PSU in adolescents is directly related to poor sleep quality and that bedtime procrastination indirectly affects sleep quality through a mediating effect. Both high levels of smartphone use and the associated bedtime procrastination can lead to sleep problems such as shorter sleep duration, difficulty in falling asleep, and insomnia.^44,45^ This study confirms that bedtime procrastination plays a mediating role, as previous literature has shown a connection between bedtime procrastination and low sleep quality.^23,25,46^ The literature explains bedtime procrastination associated with PSU with the nature of phone applications designed to keep users for a long time, losing time awareness while using the smartphone and taking time for oneself by using the smartphone after a busy day, smartphone use affects circadian rhythm and increased arousal pathways before sleep.^14,15,19,47^ The indirect effects of bedtime procrastination behavior, a less explored topic in adolescents than adults, could guide future research examining mediating variables influencing sleep quality in adolescents.

This study has some limitations. This study used self-report measures that may not accurately identify the participants’ actual behavior. Future research should use objective tools such as actigraphy or applications that provide information about smartphone use to obtain more accurate sleep and phone use data. Since PSU is associated with general procrastination, it may cause adolescents to postpone tasks from day to night, which may have consequences such as postponing bedtime and affecting sleep quality.^48^ Future studies can investigate the mediating role of general procrastination behaviors during the day on sleep in adolescents. The cross-sectional design of the study prevents causal conclusions. Therefore, in future studies, the mediating effect of other variables, such as self-regulation in adolescents, including sociodemographic variables in the analysis, and using methods such as longitudinal research design may provide causal results related to smartphones and sleep. Our study evaluated the relationships between smartphone use and sleep quality in adolescents during the school period. There may be changes in these relationships during vacation periods. It may be helpful for future studies to consider weekends or long vacation periods.

Findings from this study confirm that smartphone use negatively affects adolescents’ sleep quality. The results also indicate that bedtime procrastination may have a mediating role in this relationship. To the best of our knowledge, there is no study on the mediating effect of bedtime procrastination and the frequency and activity type of smartphone use before bedtime in Turkish adolescents. The findings of this study suggest that interventions such as sleep hygiene targeting bedtime procrastination behavior may help improve the sleep quality of adolescents with PSU signs. Additionally, the results highlight the significance of encouraging healthy tech habits and reducing technology use to improve sleep quality in adolescents.

## Figures and Tables

**Table 1.** Sociodemographic Characteristics of Participant

Characteristics	n (%)
Age	3
13	48 (19.6)
14	40 (16.3)
15	34 (13.9)
16	57 (23.3)
17	44 (18)
18	22 (9)
Sex	3
Girl	145 (59.2)
Boy	100 (40.8)
Smartphone use	3
Less than 1 hour	50 (20.4)
Between 1 and 2 hours	65 (26.5)
Between 2 and 3 hours	47 (19.2)
Between 3 and 4 hours	41 (16.7)
Between 4 and 5 hours	38 (15.5)
More than 5 hours	4 (1.6)
Other technological devices use	3
Less than 1 hour	123 (50.2)
Between 1 and 2 hours	58 (23.7)
Between 2 and 3 hours	32 (13.1)
Between 3 and 4 hours	16 (6.5)
Between 4 and 5 hours	13 (5.3)
More than 5 hours	3 (1.2)
Use smartphone in bed	3
Yes	229 (93.5)
No	16 (6.5)
Days per week using smartphone in bed 3	
0	16 (6.5)
1	32 (13.1)
2	18 (7.3)
3	17 (6.9)
4	13 (5.3)
5	3 (1.2)
6	9 (3.7)
7	137 (55.9)
Time spent using a smartphone in bed 3	
0	16 (6.5)
Less than 15 minutes	55 (22.4)
Between 16 and 30 minutes	71 (29)
Between 31 and 60 minutes	44 (18)
More than 60 minutes	59 (24.1)
Smartphone activity in bed	3
Phone calling	88 (35.9)
Messaging	132 (53.5)
Social media	188 (76.7)
Internet browsing	126 (51.4)
Video watching	167 (68.2)
Internet game playing	93 (38)
Other	63 (25.7)
Problematic smartphone use	3
Yes	112 (46.9)
No	233 (53.1)

**Table 2.** Descriptive Statistics of the Study Variables

Variables	Mean	SD
Smartphone use	2.85	1.41
Other technological devices use	1.96	1.25
Days per week using smartphone in bed	4.89	2.63
Time spent using a smartphone in bed	2.31	1.22
Total number of activities with a smartphone in bed	3.49	2.11
Use of all technological devices	4.82	2.07
Bedtime procrastination (BP)	26.51	7.34
Problematic smartphone use (SAS-SV)	29.61	11.92
Sleep quality (ASWS)	3.15	0.74

ASWS, Adolescent Sleep–Wake Scale; BP, bedtime procastination; SAS-SV, Smartphone Addiction Scale-Short Version.

**Table 3.** Descriptive Statistics and Differences Between the Gender

Variables	Girl		Boy		*t*	*P*	Cohen’s *d*
	Mean	SD	Mean	SD			
Smartphone use	3	1.47	2.75	1.37	1.335	.183	1.412
Other technological devices use	1.73	1.17	2.13	1.28	-2.530	.012^*^3	1.240
Days per week using smartphone in bed	4.83	2.56	4.94	2.69	−0.337	.736	2.644
Time spent using a smartphone in bed	2.3	1.23	2.33	1.22	−0.194	.846	1.229
Total number of activities with a smartphone in bed	3.54	2.14	3.46	2.09	0.257	.797	2.116
Use of all technological devices	4.73	2.05	4.88	2.09	−0.568	.571	2.077
Bedtime procrastination (BP)	26.32	8.1	26.64	6.79	−0.329	.742	7.362
Problematic smartphone use (SAS-SV)	31.35	12.54	28.41	11.37	1.872	.063	11.865
Sleep quality (ASWS)	3.16	0.68	3.15	0.78	0.135	.893	0.745

ASWS, Adolescent Sleep–Wake Scale; BP, bedtime procastination; SAS-SV, Smartphone Addiction Scale-Short Version.

*P < .05.

**Table 4.** Correlation Analysis Between Variables

Scales		1	2	3	4	5	6	7	8	9	10
Age 3	*r*3	1	.093	.001	.114	.093	−.053	.052	-.043	.056	.064
	*P*3	3	.147	.989	.076	.146	.409	.415	.508	.384	.318
Smartphone use 3	*r*3	.093	1	.205**	.427**	.466**	−.277**	.390**	.217**	.393**	.806**
	*P*3	.147	3	**.001**3	**.000**3	**.000**3	**.000**3	**.000**3	**.001**3	**.000**3	**.000**3
Other technological devices use 3	*r*3	.001	.205**	1	.174**	.177**	−.270**	.071	.172**	.182**	.744**
	*P*3	.989	**.001**3	3	**.006**3	**.005**3	**.000**3	.269	**.007**3	**.004**3	**.000**3
Days per week using smartphone in bed 3	*r*3	.114	.427**	.174**	1	.639**	−.312**	.421**	.314**	.533**	.396**
	*P*3	.076	**.000**3	**.006**3	3	**.000**3	**.000**3	**.000**3	**.000**3	**.000**3	**.000**3
Time spent using a smartphone in bed 3	*r*3	.093	.466**	.177**	.639**	1	−.354**	.421**	.319**	.514**	.425**
	*P*3	.146	**.000**3	**.005**3	**.000**3	3	**.000**3	**.000**3	**.000**3	**.000**3	**.000**3
Sleep quality 3	*r*3	−.053	−.277**	−.270**	−.312**	−.354**	1	−.435**	−.628**	−.261**	−.353**
	*P*3	.409	**.000**3	**.000**3	**.000**3	**.000**3	3	**.000**3	**.000**3	**.000**3	**.000**3
Problematic smartphone use 3	*r*3	.052	.390**	.071	.421**	.421**	−.435**	1	.443**	.393**	.309**
	*P*3	.415	**.000**3	.269	**.000**3	**.000**3	**.000**3	3	**.000**3	**.000**3	**.000**3
Bedtime procrastination 3	*r*3	−.043	.217**	.172**	.314**	.319**	−.628**	.443**	1	.243**	.252**
	*P*3	.508	**.001**3	**.007**3	**.000**3	**.000**3	**.000**3	**.000**3	3	**.000**3	**.000**3
Total number of activities with a smartphone in bed 3	*r*3	.056	.393**	.182**	.533**	.514**	−.261**	.393**	.243**	1	.379**
	*P*3	.384	**.000**3	**.004**3	**.000**3	**.000**3	**.000**3	**.000**3	**.000**3	3	**.000**3
Use of all technological devices 3	*r*3	.064	.806**	.744**	.396**	.425**	−.353**	.309**	.252**	.379**	1
	*P*3	.318	**.000**3	**.000**3	**.000**3	**.000**3	**.000**3	**.000**3	**.000**3	**.000**3	3

Bold values indicate statistical significance.

**P ≤ .001.

**Table 5.** The Mediating Effect of Bedtime Procrastination on the Relationship Between Problematic Smartphone Use and Sleep Quality in Adolescents

Effect	*β*	*t*	*P*	95% CI	
				Lower Limit	Upper Limit
a: PSU ⟶ BP	0.44	7.68	<.001	0.20	0.34
b: BP ⟶ SQ	−0.54	−9.93	<.001	−0.06	−0.04
c^t^: PSU ⟶ SQ	−0.44	−7.54	<.001	−0.03	−0.02
c^d^: PSU ⟶ SQ	−0.20	−3.60	<.001	−0.02	−0.01
a*b (indirect)	−0.24	3	3	−0.02	−0.10

c^d^, direct effect; c^t^, total effect; *β*, standardized coefficients; PSU, problematic smartphone use; BP, bedtime procrastination; SQ, sleep quality
